# Stabilizing Extreme Few‐Shot ECG Classification via Self‐Supervised Contrastive Pretraining

**DOI:** 10.1111/anec.70188

**Published:** 2026-04-12

**Authors:** LiuPing Zeng, JingMei Pan, YangJie Lu, Xian Pan

**Affiliations:** ^1^ The First Affiliated Hospital of Jinan University Guangzhou China; ^2^ Universiti Malaya Kuala Lumpur Malaysia; ^3^ Guangzhou College of Technology and Business Guangzhou China

**Keywords:** electrocardiogram, extreme few‐shot learning, optimization collapse, self‐supervised contrastive learning, training stability

## Abstract

In clinical electrocardiogram (ECG) analysis, high‐quality annotations are expensive and difficult to scale, leaving many tasks in an extreme few‐shot learning regime. We formulate a single‐label Top‐5 rhythm classification task on PTB‐XL and strictly limit supervised training to *N* = 70 labeled samples (14 per class) to characterize failure modes of scratch training and assess the stabilizing effect of self‐supervised learning (SSL). We first perform SimCLR‐style contrastive pretraining with the NT‐Xent loss on 16,304 unlabeled ECG recordings, followed by supervised fine‐tuning. To isolate the independent contribution of SSL initialization, downstream augmentation is disabled (NoAug) in the core evaluation. Performance and stability are assessed using Macro‐F1, best validation epoch, and a collapse rate defined as Best Epoch ≤ 1. Under *N* = 70, scratch training exhibits systematic early collapse (Macro‐F1 = 0.115 ± 0.072; collapse rate = 66.7%), which is not alleviated by strong downstream augmentation alone (0.108 ± 0.042; 66.7%). In contrast, SSL fine‐tuning without downstream augmentation improves Macro‐F1 to 0.192 ± 0.003 and reduces the collapse rate to 0%, with markedly reduced inter‐seed variance. A balanced *N* = 300 reference achieves Macro‐F1 = 0.297 ± 0.018. These results indicate that, in extreme few‐shot ECG classification, the primary bottleneck is training reproducibility rather than peak accuracy, and SSL contrastive pretraining provides a robust initialization that substantially mitigates collapse and improves usability.

## Graphical abstract

Under extreme few‐shot ECG classification, supervised training frequently collapses to degenerate solutions. Self‐supervised pretraining stabilizes optimization, eliminates early collapse, and enables reproducible multi‐class rhythm classification with limited labeled data.

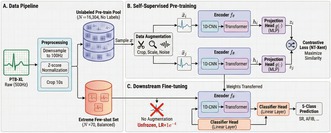

## 1 Introduction

Cardiovascular disease (CVD) remains a leading cause of morbidity and mortality worldwide, continuing to impose a substantial and growing population‐level burden (Global Burden of Cardiovascular Diseases and Risks 2023 Collaborators 2025). Contemporary epidemiological summaries further highlight the scale of CVD impact and its persistent contribution to premature death and disability (Martin et al. 2024). Electrocardiography (ECG) is a key diagnostic modality in cardiovascular care, including for rhythm disorders such as atrial fibrillation/flutter, and expanding access to screening and diagnostic technologies is important as the burden of these conditions rises (Hindricks et al. 2021). Accordingly, automated detection and classification of ECG abnormalities—including arrhythmia‐related rhythm disorders—has become an active and widely adopted application area for machine learning and deep learning, as exemplified by large‐scale multi‐institutional benchmarking efforts such as the PhysioNet/Computing in Cardiology Challenge 2021 (Reyna et al. 2022).

With advances in deep neural networks for one‐dimensional signals—particularly convolutional and residual architectures—deep learning has demonstrated strong performance for automated 12‐lead ECG diagnosis and classification when trained on large, systematically labeled datasets, achieving high F1 scores and very high specificity on held‐out test sets (Ribeiro et al. 2020). Representative studies have shown that end‐to‐end deep neural networks can achieve cardiologist‐competitive performance for arrhythmia detection and classification when evaluated on independent test sets annotated by board‐certified cardiologists under consensus protocols (Hannun et al. 2019). Large, curated clinical programs have also demonstrated robust deep‐learning performance for 12‐lead ECG diagnosis when trained with sufficiently extensive labeled data. Beyond rhythm‐focused applications, recent work shows that AI models trained on large, diverse ECG–imaging cohorts can use ECGs to screen for structural heart disease, with consistent performance across care settings and demographic groups, and validation in external cohorts and prospective evaluation (Poterucha et al. 2025).

However, benchmark‐style evaluations can give an overly reassuring picture of deployability: reported performance depends critically on how development and evaluation data are defined, separated, and how well they represent the intended clinical population; when data quality, representativeness, or outcome assessment procedures differ across settings or subgroups, real‐world performance may not match benchmark results (Collins et al. 2024). In routine clinical AI development, model performance is highly sensitive to how data and labels are defined and collected; incomplete or non‐transparent reporting can yield overly optimistic results due to issues such as skewed data selection or data leakage, underscoring the need to clearly document datasets and training/validation procedures for reproducibility and clinical validity (Kolbinger et al. 2024). As a result, a common starting point for practical ECG AI in new hospitals, devices, or task definitions is a low‐resource regime with only tens to a few hundreds of reliable labeled cases. More importantly, in extreme few‐shot settings (e.g., only ~10–20 labeled examples per class), the challenge is not merely a gradual decrease in average metrics or a simple increase in overfitting risk. A more deployment‐critical failure mode is training instability: outcomes can become highly sensitive to random initialization and optimization noise, sometimes collapsing early into near‐constant predictors with little meaningful learning progress. In medical AI, unpredictable and hard‐to‐anticipate failure modes threaten safe and reproducible deployment; CONSORT‐AI therefore emphasizes transparent reporting to enable independent evaluation and replication, including clear documentation of algorithm versioning, input‐data handling, and systematic analysis of performance errors, alongside post‐deployment surveillance (Liu et al. 2020).

Self‐supervised pre‐training—particularly contrastive learning—provides a practical way to leverage abundant unlabeled physiological signals, alleviating the need for extensive high‐quality clinical labels by learning representations that transfer effectively to downstream tasks via linear evaluation and fine‐tuning (Kiyasseh et al. 2021). Contrastive learning provides a simple mechanism for representation learning by maximizing agreement between two differently augmented views of the same example, while using the other augmented samples in the minibatch as negatives to separate representations in latent space (Chen et al. 2020). Nevertheless, from a clinical translation perspective, a frequently under‐addressed question is whether SSL's primary value in the extreme few‐shot regime lies in boosting mean performance or in improving trainability and run‐to‐run reproducibility. This ambiguity is compounded because observed gains can be confounded by sampling bias, class‐imbalance effects, and downstream augmentation strength, making it difficult to attribute improvements causally.

To address this gap, we focus on stabilizing extreme few‐shot ECG rhythm classification using an audit‐ready, mechanism‐isolating design on the PTB‐XL dataset (Wagner et al. 2020). PTB‐XL offers a large, publicly accessible clinical 12‐lead ECG resource, and published benchmarking results with well‐defined evaluation procedures enable more controlled and comparable comparisons of ECG analysis algorithms across tasks and experimental settings (Strodthoff et al. 2020). We study five clinically common rhythm classes (SR, AFIB, STACH, SARRH, SBRAD) and compress supervised training to an extreme few‐shot setting (*N* = 70; 14 per class) to emulate realistic label‐scarce starting conditions. Methodologically, we employ SimCLR‐style contrastive pretraining on unlabeled ECGs and then transfer the pretrained encoder to the downstream five‐class task via low–learning‐rate fine‐tuning. Methodologically, we employ SimCLR‐style contrastive pretraining on unlabeled ECGs and then transfer the pretrained encoder to the downstream five‐class task via low–learning‐rate fine‐tuning. Our central contribution is not adding complexity, but enforcing a strict upper‐bound/lower‐bound/mechanism‐ablation control system that isolates gains attributable to SSL initialization. We further quantify “failure under extreme few‐shot” beyond static metrics by introducing a training‐dynamics proxy (best validation epoch) and a collapse‐rate definition that operationalizes early optimization collapse as auditable evidence.

Overall, this study makes three contributions. First, we show that in PTB‐XL extreme few‐shot rhythm classification, scratch supervised training exhibits a systematic trainability failure with consistent dynamical signatures rather than merely lower mean scores. Second, we demonstrate that SSL initialization primarily improves optimization reachability and reproducibility—suppressing early collapse and reducing inter‐seed variance—thereby turning extreme few‐shot training from a high‐risk event into a repeatable procedure. Third, through augmentation‐only and SSL‐with/without‐augmentation ablations, we delineate the boundary of downstream strong augmentation in the extreme few‐shot regime, showing that augmentation alone is insufficient to replace representation pretraining and may introduce additional instability.

## 2 Materials and Methods

### 2.1 Dataset, Task Definition, and Controlled Experimental Design

**FIGURE 1:**
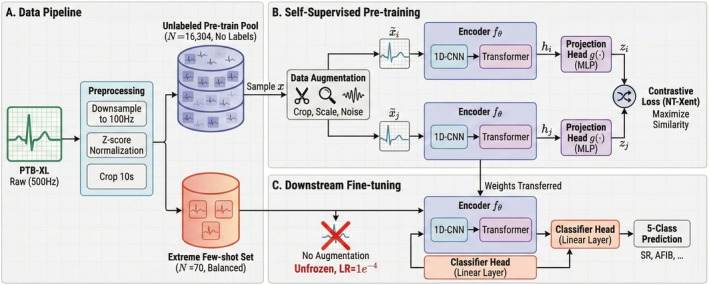
Overview of the framework for stabilizing extreme few‐shot ECG rhythm classification. (A) Data pipeline. Raw 12‐lead ECG recordings from PTB‐XL (500 Hz, 10 s) are downsampled to 100 Hz and normalized using per‐lead *Z*‐score normalization, forming an unlabeled pretraining pool (*N* = 16,304) and an extreme few‐shot labeled set (*N* = 70, class‐balanced). (B) Self‐supervised contrastive pretraining. Unlabeled ECG signals are augmented to generate paired views via random cropping/resampling, amplitude scaling, and noise injection. A CNN–Transformer encoder with a projection head is trained using the NT‐Xent contrastive objective to learn transferable representations. (C) Stabilized downstream fine‐tuning. The pretrained encoder is transferred to a five‐class rhythm classification task and fine‐tuned with full unfreezing and a low learning rate (1 × 10^−4^). In the core setting, downstream data augmentation is disabled (NoAug) to strictly isolate the contribution of self‐supervised initialization, while augmentation‐enabled variants are included as ablation controls.

We conducted all experiments on the publicly available PTB‐XL electrocardiography dataset (Wagner et al. 2020). PTB‐XL contains 21,837 clinical 12‐lead ECG records (10 s, 500 Hz) from 18,885 subjects. To disentangle the effects of data budget, sampling balance, downstream augmentation, and self‐supervised initialization under extreme label scarcity, we organized the study into a controlled pipeline spanning data construction, contrastive pretraining, and downstream fine‐tuning. An overview of the full workflow and controlled comparisons is provided in Figure 1.

Deep neural models have been shown to perform competitively on 12‐lead ECG interpretation when large‐scale labeled data are available (Ribeiro et al. 2020). We formulated a single‐label rhythm classification task using five clinically common rhythm classes with relatively stable annotations: sinus rhythm (SR), atrial fibrillation (AFIB), sinus tachycardia (STACH), sinus arrhythmia (SARRH), and sinus bradycardia (SBRAD). To emulate an “extreme label scarcity” regime and to attribute performance gains to a testable causal chain, we adopted an upper‐bound/lower‐bound/mechanism‐isolation design. Specifically, Groups A and A' served as 300‐sample supervised references, where A followed the original sampling distribution and A' enforced strict class balance, to characterize an upper‐bound regime under relatively sufficient labels. Group C represented the extreme few‐shot baseline (*N* = 70, class‐balanced; 14 samples per class) trained from scratch to expose the failure modes of supervised learning in this regime. Group E (“Aug Only”) applied strong downstream augmentation to the same *N* = 70 scratch setting to test whether augmentation alone can rescue extreme few‐shot training.

Group D was our proposed strategy (contrastive SSL pretraining + downstream fine‐tuning), reported under both downstream augmentation enabled (D, Aug) and disabled (D, NoAug). The D, NoAug configuration was treated as the core setting to strictly isolate the independent contribution of SSL initialization.

### 2.2 Signal Preprocessing and Data Splits

Each ECG record consists of 12 leads sampled at 500 Hz over a 10 s interval. To reduce computational cost while fixing the input dimensionality, all records were resampled to 100 Hz and the 10 s segment was retained, yielding an input tensor:

$$X\in R^{12\times 1000}$$

To mitigate inter‐subject and inter‐lead amplitude variability, we applied per‐lead *Z*‐score normalization within each 10 s record:

$${\tilde{x}}_{l,t}=\frac{x_{l,t}-\mu_{l}}{\sigma_{l}+\epsilon }$$

where $\mu_{l}$ and $\sigma_{l}$ are the mean and standard deviation of lead l over the 10 s segment, and ϵ is a numerical stabilizer. For SSL pretraining, we constructed an unlabeled pool $D_{\text{unlabeled}}$ with 16,304 ECG records by discarding labels and retaining only waveforms.

For supervised learning, we used an extreme few‐shot labeled set $D_{\mathrm{few}}$ (*N* = 70, class‐balanced; 14 per class) and additional 300‐sample reference training sets for the upper‐bound controls (A/A′/B). All models were evaluated on an independent test set $D_{\text{test}}$ that preserves the real‐world long‐tailed rhythm distribution, with 2035 records in total.

### 2.3 Self‐Supervised Contrastive Pretraining

We performed contrastive SSL pretraining using a SimCLR‐style framework on $D_{\text{unlabeled}}$ without using any label information. For each ECG signal x, we sampled two random augmentations $t,t^{\prime }∼T$ to generate two views:

$${\tilde{x}}_{i}=t\left(x\right),{\tilde{x}}_{j}=t^{\prime }\left(x\right)$$

The augmentation family T included random cropping/resampling, amplitude scaling, and additive Gaussian noise, intended to model heart‐rate variability, amplitude drift, and acquisition noise.

The encoder $f_{\theta }\left(\cdot \right)$ produced a representation $h=f_{\theta }\left(\tilde{x}\right)$, and a projection head $g_{\phi }\left(\cdot \right)$ mapped h into the contrastive space $z=g_{\phi }\left(h\right)$. The projection head was a two‐layer MLP (64 → 128 → 64) with ReLU activations.

We optimized the normalized temperature‐scaled cross‐entropy (NT‐Xent) objective.

Given a batch of N original signals and 2N augmented views, the loss for a positive pair $\left(i,j\right)$ was

$$L_{i,j}=-\log\frac{\exp\left(\frac{\mathrm{sim}\left(z_{i},z_{j}\right)}{\tau }\right)}{\sum_{k=1}^{2N}1_{\left[k\neq i\right]}\exp\left(\frac{\mathrm{sim}\left(z_{i},z_{k}\right)}{\tau }\right)}$$

where $\mathrm{sim}\left(u,v\right)=\frac{u^{⊤}v}{∥u∥∥v∥}$ is cosine similarity and τ is the temperature (set to 0.1).

After pretraining, we discarded the projection head $g_{\phi }$ and transferred only the pretrained encoder parameters to downstream training. We note that contrastive SSL has been shown to be particularly well‐suited for cardiac signals, supporting transferable representations under limited labels.

### 2.4 Downstream Fine‐Tuning and Evaluation Metrics

For the downstream task, we attached a linear classification head $W\in {\mathbb{R}}^{64\times 5}$ to the pretrained encoder.

Given an input x, the encoder output was $h=f_{\theta }\left(x\right)$, and class probabilities were computed as

$$\hat{y}=\text{softmax}\left(W^{⊤}h\right)$$

We optimized the multiclass cross‐entropy objective.

$$L_{\mathrm{CE}}=-\sum_{c=1}^{5}y_{c}\log\left({\hat{y}}_{c}\right)$$

where y is a one‐hot label vector.

To improve optimization stability in the extreme few‐shot regime, we fine‐tuned the pretrained encoder end‐to‐end (full unfreezing) using a low learning rate (1 × 10^−4^) to limit excessive parameter updates and reduce representation drift.

In the core isolation setting D (NoAug), we disabled all downstream augmentation during fine‐tuning, while E and D (Aug) served as ablation controls.

All experiments followed a consistent train–validation–test procedure with early stopping on the validation set (patience = 10), selecting the best epoch and reporting results on $D_{\text{test}}$.

Primary metrics were Accuracy and Macro‐F1, where for class c,

$$P_{c}=\frac{TP_{c}}{TP_{c}+FP_{c}},R_{c}=\frac{TP_{c}}{TP_{c}+FN_{c}},F1_{c}=\frac{2P_{c}R_{c}}{P_{c}+R_{c}}$$

and

$$\text{Macro}\;F1=\frac{1}{5}\sum_{c=1}^{5}F1_{c}$$

To reflect the high variance of extreme few‐shot training, we repeated each experiment with three random seeds and reported mean ± standard deviation.

We further operationalized training collapse to quantify optimization instability using the best validation epoch $e^{*}$.

A run was considered collapsed if $e^{*}\leq 1$, and the collapse rate was defined as

$$\text{CollapseRate}=\frac{1}{R}\sum_{r=1}^{R}1\left[e_{r}^{*}\leq 1\right],R=3$$

## 3 Results

### 3.1 Stable Few‐Shot ECG Classification

Table 1 summarizes test‐set Macro F1, Accuracy, best validation epoch (Best Epoch), and collapse rate for all experimental groups. Under relatively sufficient supervision (*N* = 300), the reference groups A (imbalanced sampling), A′ (strictly balanced sampling), and B (300 samples with augmentation) all exhibited stable and reproducible training behavior. Their Macro F1 scores were 0.319 ± 0.019, 0.297 ± 0.018, and 0.318 ± 0.024, respectively, with zero collapse cases across random seeds. These results indicate that, when labels are moderately abundant, the lightweight CNN–Transformer architecture can converge reliably across different random initializations, with sustained performance improvement over training (Best Epoch ≈ 19–44).

**TABLE 1:** Performance and stability metrics across experimental groups on the independent test set.

Experimental group	*N*	Training strategy	Downstream augmentation	Macro F1 (mean ± SD)	Accuracy (mean ± SD)	Best epoch (mean)	Collapse rate
A: 300 (Imbal)	300	Supervised learning (from scratch)	Disabled	0.319 ± 0.019	0.775 ± 0.032	21.3	0.0%
A′: 300 (Bal)	300	Supervised learning (from scratch)	Disabled	0.297 ± 0.018	0.785 ± 0.025	43.7	0.0%
B: 300 Aug	300	Supervised learning (from scratch)	Enabled	0.318 ± 0.024	0.772 ± 0.030	19.0	0.0%
C: Scratch (70)	70	Supervised learning (from scratch)	Disabled	0.115 ± 0.072	0.294 ± 0.252	1.7	66.7%
E: Aug Only (70)	70	Supervised learning (from scratch)	Enabled	0.108 ± 0.042	0.206 ± 0.128	1.7	66.7%
D: SSL (70, Aug)	70	SSL pretraining + fine‐tuning	Enabled	0.162 ± 0.049	0.281 ± 0.075	18.0	33.3%
D: SSL (70, NoAug)	70	SSL pretraining + fine‐tuning	Disabled	0.192 ± 0.003	0.331 ± 0.044	40.3	0.0%

*Note:* Values are reported as mean ± SD over three random seeds. Collapse rate is defined as the proportion of runs with Best Epoch ≤ 1. “Downstream augmentation” refers to strong data augmentation applied during supervised fine‐tuning; contrastive augmentation during SSL pretraining is used in all SSL‐based groups.

When the number of labeled training samples was reduced to *N* = 70 (14 per class), the nature of the problem fundamentally changed. The scratch‐trained model (Group C) did not merely suffer a gradual performance degradation, but instead exhibited a characteristic pattern of systematic training failure. Its Macro F1 dropped to 0.115 ± 0.072, while Accuracy showed extremely large variance (0.294 ± 0.252). The mean Best Epoch was only 1.7, accompanied by a collapse rate of 66.7% (Table 1). In this regime, model outcomes were highly dependent on random initialization and optimization noise: under the same configuration, changing the random seed could shift the result from marginally usable to a nearly constant predictor. Such brittleness and poor generalisability undermine clinical applicability: performance can deteriorate under real‐world dataset shift, motivating robust clinical evaluation, independent representative test sets for comparison, and regulatory quality control with post‐deployment surveillance to ensure safe use (Kelly et al. 2019).

**FIGURE 2:**
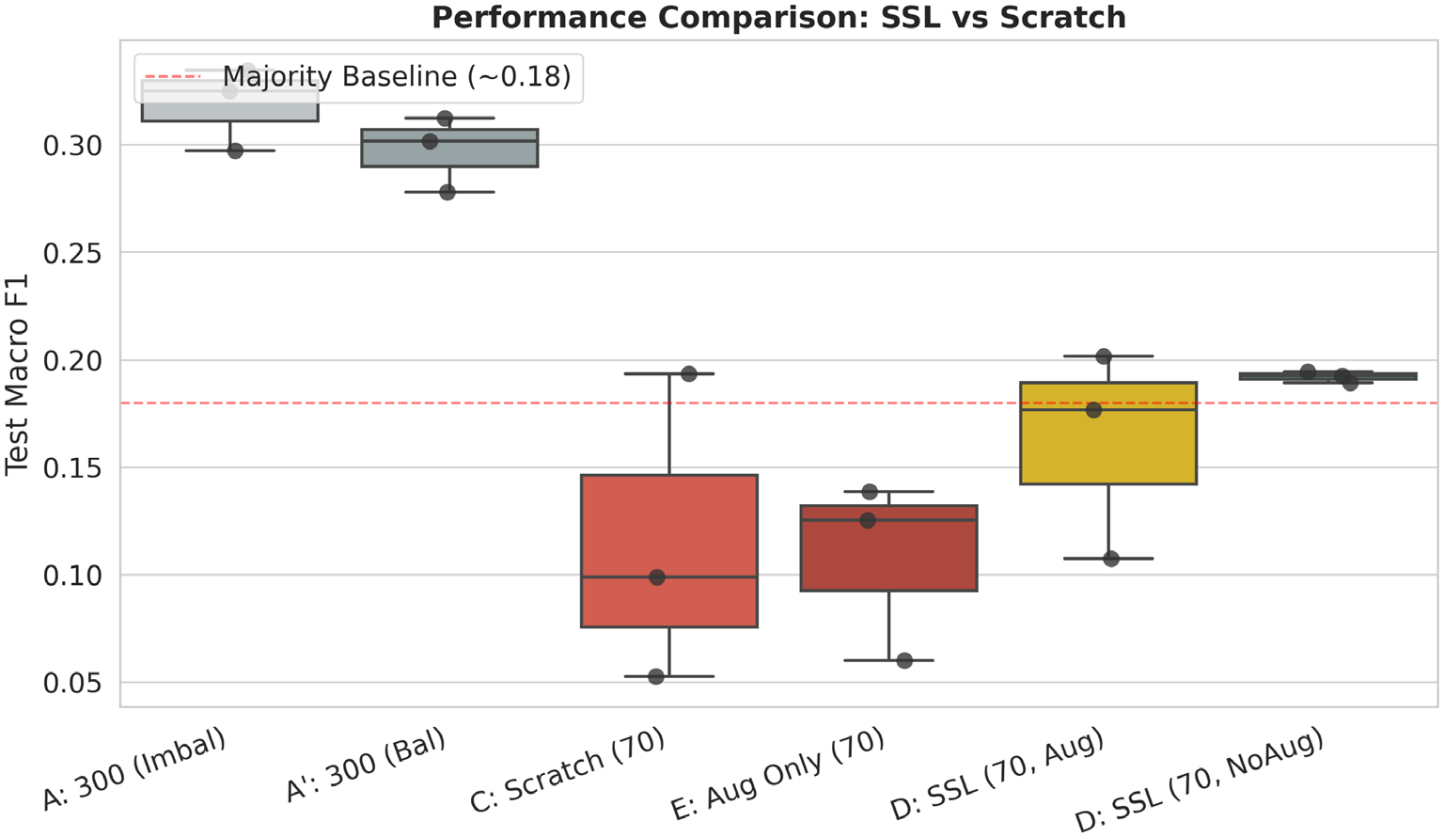
Impact of self‐supervised pretraining and data balance on few‐shot ECG classification performance. Boxplots show the distribution of test Macro F1 across training regimes (*n* = 3 random seeds). The dashed red line denotes the majority‐class baseline (~0.18), representing a degenerate predictor. Groups A and A′ (*N* = 300) provide upper‐bound references under relatively sufficient supervision. In contrast, Groups C (Scratch, *N* = 70) and E (Aug Only, *N* = 70) illustrate characteristic failure modes under extreme few‐shot conditions, with low medians and large inter‐seed variability. SSL‐based fine‐tuning (Group D), particularly without downstream augmentation (D, NoAug), achieves higher Macro F1 while markedly reducing inter‐seed variance, indicating improved robustness and reproducibility under severe label scarcity.

Under the same *N* = 70 constraint, initializing the model with self‐supervised pretraining and disabling downstream augmentation during fine‐tuning (D: SSL, NoAug) substantially altered the training dynamics. Macro F1 increased to 0.192 ± 0.003 and Accuracy to 0.331 ± 0.044, while inter‐seed variance was almost eliminated and the collapse rate dropped to 0%. The implications of this shift are illustrated in Figures 2 and 6: the distribution of D: SSL (70, NoAug) not only dominates that of scratch training but also consistently exceeds the majority‐class baseline (~0.18), indicating a transition from frequent training failure to stable and effective learning under extreme label scarcity. This pattern is consistent with prior ECG studies showing that self‐supervised learning can improve robustness and label efficiency under limited supervision.

Quantitatively, the Macro F1 achieved by D: SSL (70, NoAug) corresponds to approximately 64.6% of the balanced *N* = 300 upper‐bound reference (A′; 0.192/0.297), despite using only 23% of the labeled data (70 vs. 300). This result demonstrates that SSL can recover a substantial fraction of discriminative capability with drastically fewer labels. More importantly, it yields reproducible training outcomes, which constitutes the primary practical value of SSL in extreme few‐shot medical AI, where reliability and deployability are often more critical than absolute peak performance.

### 3.2 Optimization Dynamics: Early Collapse Under Scratch Training and Stabilization by SSL

**FIGURE 3:**
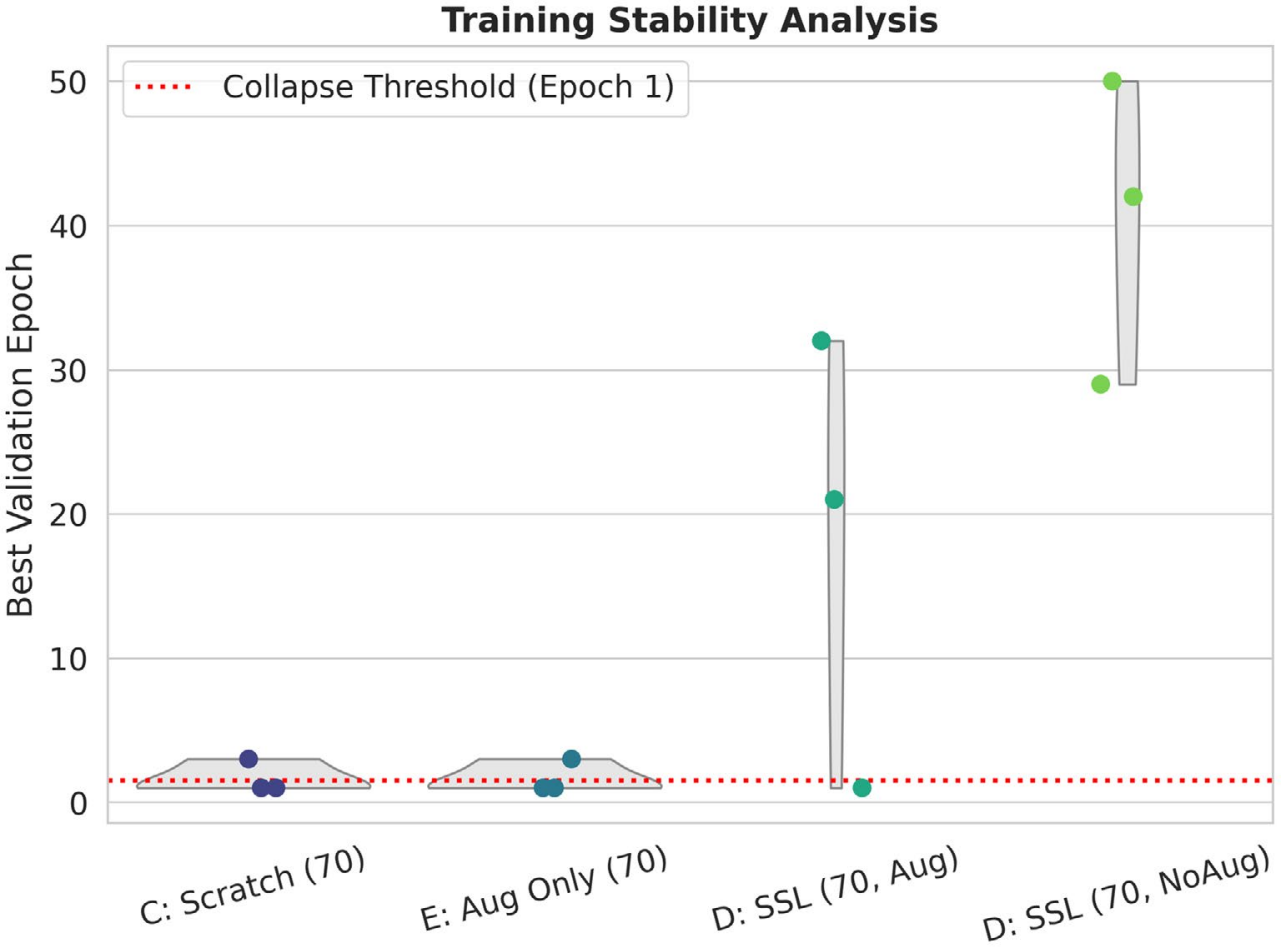
Training stability analysis using the best validation epoch as a proxy for optimization dynamics in the extreme few‐shot regime. Plots summarize the distribution of the best validation epoch across runs (*n* = 3 random seeds) for models trained with *N* = 70 labeled samples. The dashed red line marks the collapse threshold (best_epoch = 1): Runs whose best validation performance occurs at epoch 1 are treated as early‐collapse cases, indicating failure to progress beyond a degenerate solution. Scratch training (C) and augmentation‐only training (E) concentrate near the threshold, whereas SSL initialization shifts the distribution to substantially later epochs. Notably, SSL without downstream augmentation (D, NoAug) shows consistently late best_epoch with zero collapse cases, indicating improved optimization stability and reproducibility.

Reporting only mean performance can obscure a critical failure mode in extreme few‐shot training. In our experiments at N=70, unsuccessful runs often fail non‐gradually—collapsing at the very beginning of optimization and rapidly degenerating into a near‐constant predictor—highlighting the need to characterize run‐to‐run variability by repeating training with identical settings and reporting dispersion (e.g., standard deviation) rather than a single average (Renard et al. 2020). To quantify this phenomenon, we use the best validation epoch (best_epoch) as a proxy for optimization dynamics. When best_epoch ≤ 1, the model generally fails to enter an effective representation‐learning phase: validation performance peaks immediately and shows no substantive improvement thereafter, which is consistent with a degenerate solution dominated by a single predicted class. Figure 3 summarizes the distribution of best_epoch across training regimes in the *N* = 70 setting. Scratch training (C) and augmentation‐only training (E) cluster near the collapse threshold and both exhibit a 66.7% collapse rate over three seeds (Table 1), indicating that their failure is not merely “low score” but a reproducible early‐collapse dynamic.

In contrast, SSL initialization substantially alters the optimization trajectory: D (SSL, NoAug) shifts best_epoch markedly to later stages (mean 40.3) and reduces the collapse rate to 0%, suggesting that the model can continue extracting useful signal over longer training horizons and reach genuinely improved validation optima. Notably, even with SSL initialization, strong downstream augmentation can reintroduce instability: D (SSL, Aug) still shows a 33.3% collapse rate (Table 1), indicating that under extreme label scarcity, stronger perturbations are not necessarily equivalent to improved learnability. This observation is consistent with prior ECG SSL evidence that representation quality and downstream behavior can depend critically on augmentation choices and finetuning protocols, rather than on augmentation strength per se.

**FIGURE 4:**
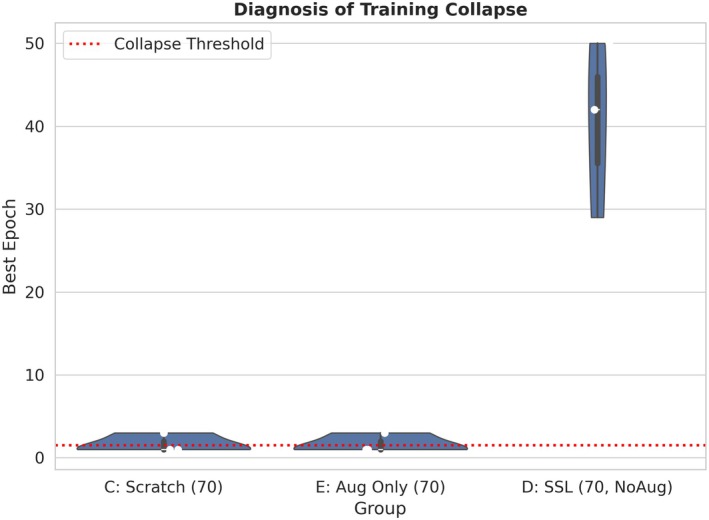
Simplified diagnosis of training collapse under extreme few‐shot supervision. Violin/box plots compare best_epoch distributions for three key regimes (*n* = 3 random seeds): Scratch training (C), augmentation‐only training (E), and SSL initialization without downstream augmentation (D, NoAug), all under *N* = 70. The dashed red line denotes the collapse threshold (best_epoch = 1). Scratch and augmentation‐only runs remain concentrated near the threshold, whereas SSL initialization shifts best_epoch to much later epochs, demonstrating a pronounced anti‐collapse effect.

To make the mechanism‐isolation argument more transparent, Figure 4 focuses on three key regimes under the same *N* = 70 protocol: C (scratch), E (Aug Only), and D (SSL, NoAug). Under identical training infrastructure, downstream augmentation alone does not prevent collapse, whereas SSL initialization—when fine‐tuned conservatively without downstream augmentation—consistently delays best_epoch to substantially later epochs. This supports our interpretation that the primary driver is a more favorable representational starting point and a more accessible optimization path, aligning with prior findings that careful finetuning (e.g., conservative schedules that avoid overwriting pretrained information) can stabilize downstream training (Mehari and Strodthoff 2022).

### 3.3 Limits of Sampling Balance and Downstream Augmentation

**FIGURE 5:**
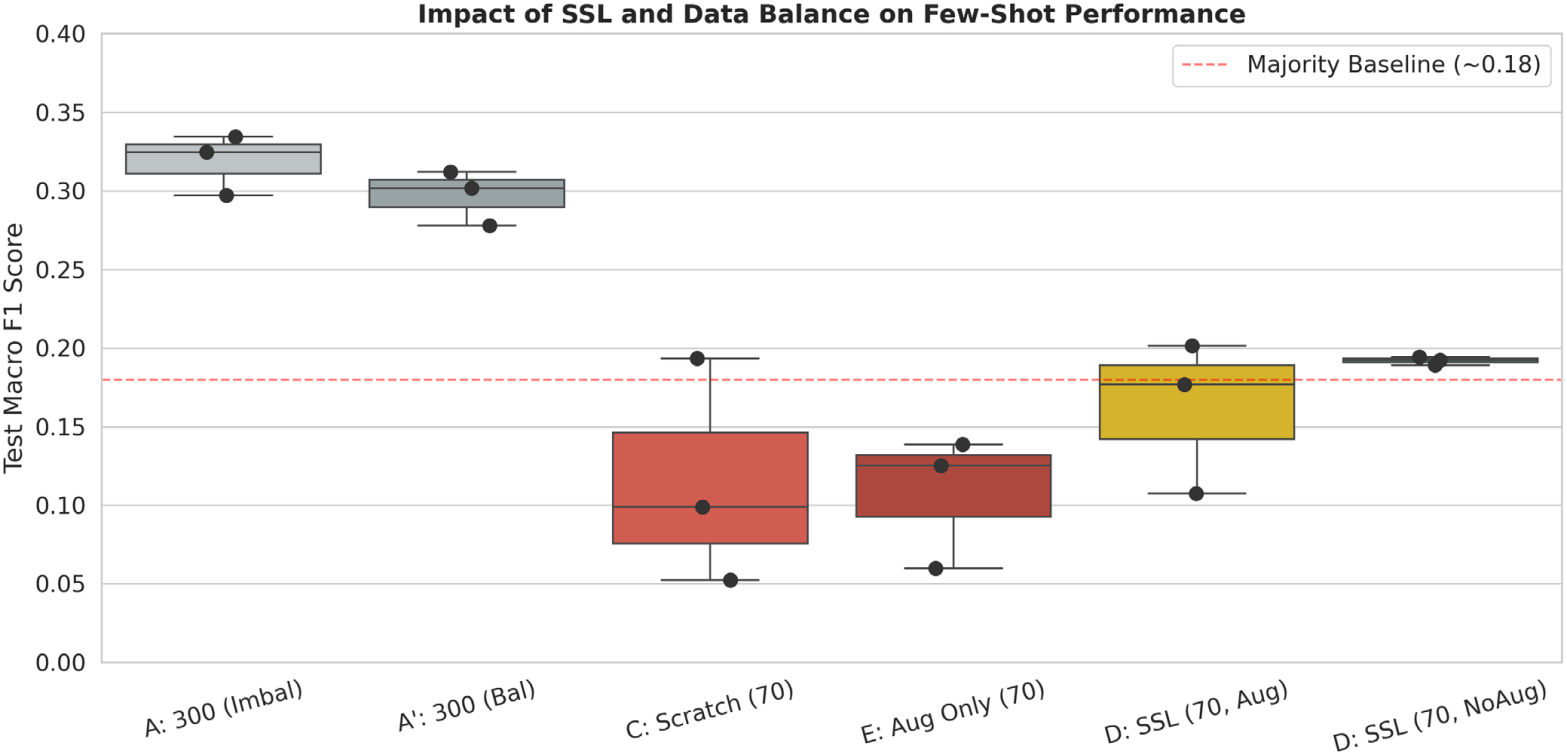
Joint effects of sampling balance, downstream augmentation, and SSL initialization on extreme few‐shot performance. Boxplots show the distribution of test Macro F1 across training regimes (*n* = 3 random seeds). The dashed red line denotes the Macro F1 of a majority‐class predictor on the test set (~0.18). Under *N* = 300, both mildly imbalanced sampling (A) and strictly balanced sampling (A′) yield stable performance with low inter‐seed variability, providing reference upper bounds under sufficient supervision. Under *N* = 70, scratch training (C) and augmentation‐only training (E) remain low and unstable, indicating that downstream augmentation alone does not recover reliable learning. SSL initialization improves performance in the few‐shot regime, and SSL fine‐tuning without downstream augmentation (D, NoAug) achieves the highest and most reproducible Macro F1 among the *N* = 70 settings.

To disentangle the effect of sampling strategy from model capacity, we evaluated mildly imbalanced sampling (A) and strict class‐balanced sampling (A′) under relatively sufficient supervision (*N* = 300), establishing a reference range for stable supervised training with the lightweight CNN–Transformer architecture. As shown in Table 1 and Figure 5, both A and A′ achieved test Macro F1 values around 0.30 with low inter‐seed variability, indicating that reproducible classifiers can be obtained when moderate label budgets are available. Notably, strictly balanced sampling (A′) yielded slightly lower Macro F1 than imbalanced sampling (A), suggesting that, in our setting, enforcing balance reshapes how different classes contribute to the training signal and optimization dynamics rather than simply making the task “easier,” consistent with prior observations that re‐balancing via re‐sampling/re‐weighting can introduce trade‐offs and does not uniformly improve performance (Cui et al. 2019).

More decisive evidence emerges in the extreme few‐shot regime (*N* = 70). Under this setting, relying solely on strong downstream augmentation (E) does not yield stable gains: its Macro F1 remains comparable to scratch training (C), and both configurations exhibit high collapse rates (Table 1). This indicates that augmentation alone does not resolve the fundamental question of whether effective learning can occur under severe label scarcity. Importantly, even after SSL initialization, enabling strong downstream augmentation (D: SSL, Aug) still results in residual instability (collapse rate 33.3%) and lower Macro F1 compared with the conservative fine‐tuning setting (D: SSL, NoAug). This boundary is consistent with the established view that data augmentation is an effective regularization strategy that can improve generalization under adequate supervision; however, in our extreme label‐scarce setting, augmentation alone does not guarantee stable trainability (Cubuk et al. 2018).

Taken together, these results indicate that under extreme few‐shot supervision, stronger data perturbations are not the determining factor for stable learning. Instead, the dominant driver of reproducible performance is the robust representational starting point provided by SSL pretraining, upon which conservative fine‐tuning without downstream augmentation yields higher and more stable performance. This delineates a clear boundary: while sampling balance provides a meaningful upper‐bound reference under sufficient labels and augmentation may help regularization in moderate regimes, neither can substitute for representation pretraining when labels are extremely scarce.

### 3.4 Class‐Wise Separability Restored by SSL

**FIGURE 6:**
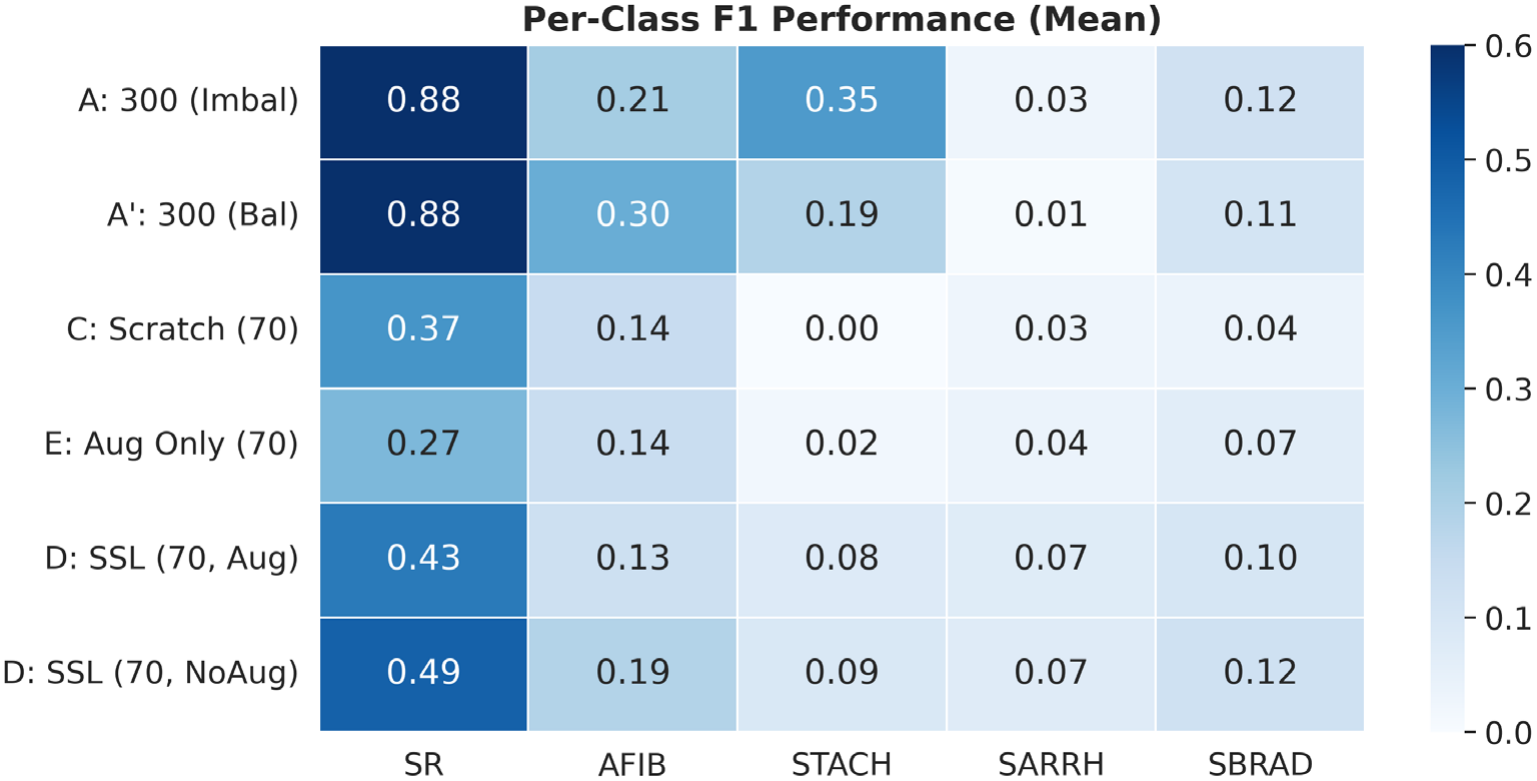
Per‐class F1 reveals restored multi‐class separability with SSL. The heatmap shows mean per‐class F1 scores across five rhythm classes (SR, AFIB, STACH, SARRH, SBRAD) for each training regime. Under *N* = 70, scratch training fails to learn discriminative representations for several classes (e.g., near‐zero F1 for STACH). In contrast, SSL initialization—particularly without downstream augmentation—consistently improves F1 across multiple classes, indicating recovery of class‐wise separability rather than reliance on a single dominant class.

To examine whether the performance gains from SSL were driven by overfitting to a single dominant class, we analyzed the mean per‐class F1 scores across rhythm categories (Figure 6).

Under the extreme few‐shot setting (*N* = 70), scratch training (C) exhibited severely impaired feature learning, with certain classes—most notably STACH—showing near‐zero F1 scores. This indicates a failure to establish meaningful decision boundaries for several rhythm types when supervision is extremely limited.

After introducing SSL initialization, D (SSL, NoAug) demonstrated systematic recovery across multiple classes, including SR, AFIB, and STACH, accompanied by a marked reduction in inter‐seed variability (Table 1). Rather than improving only a single dominant category, SSL led to coherent gains across classes, indicating restored class‐wise separability. This pattern is consistent with prior ECG evidence that pretraining on large clinical datasets and subsequent finetuning yields more stable performance and stronger discrimination in the small‐dataset regime, compared with training from scratch (Strodthoff et al. 2020).

These results support the interpretation that SSL pretraining provides a semantically richer feature manifold, shifting the downstream task from constructing class structure from scratch to aligning decision boundaries within an already informative representation space.

### 3.5 Error Patterns and Collapse Symptoms

**FIGURE 7:**
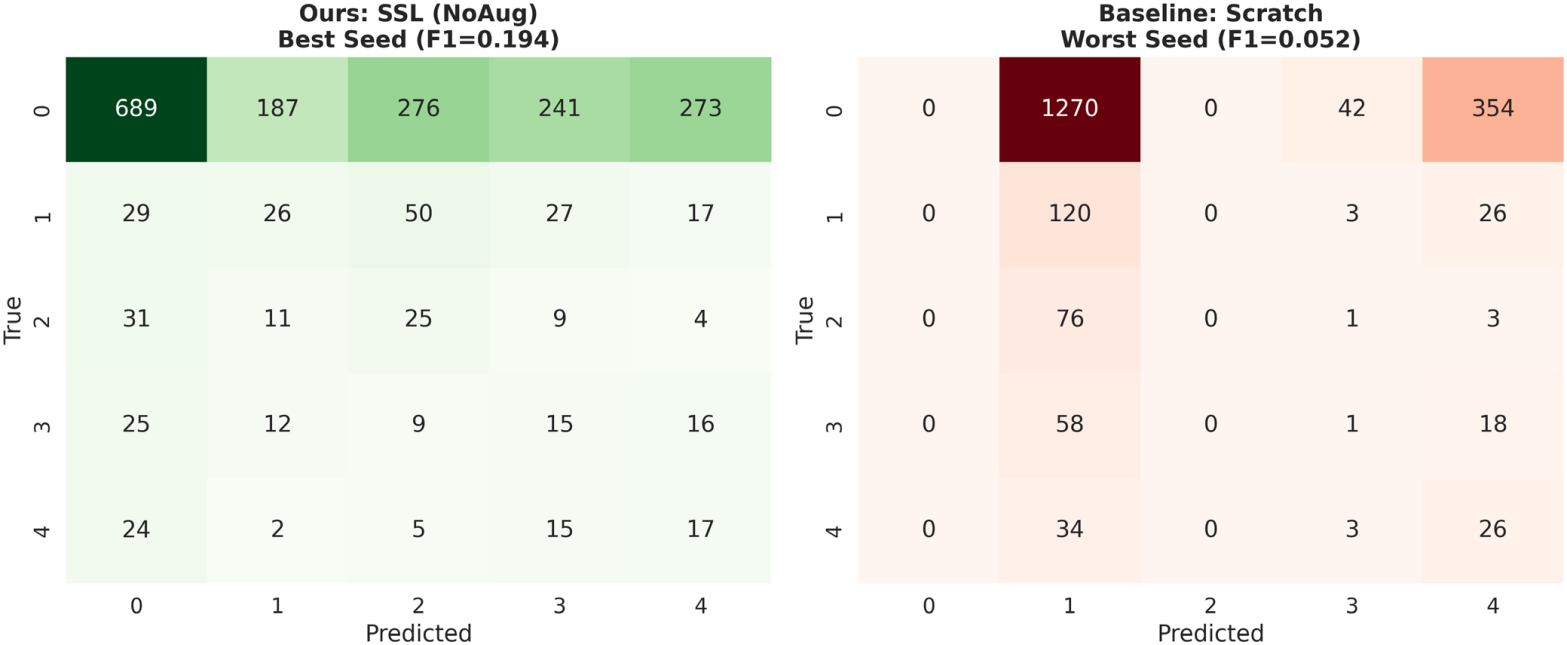
Confusion matrix evidence of collapse mitigation by SSL. Comparison between the best‐performing SSL‐initialized model without downstream augmentation (left) and the worst‐performing scratch‐trained model (right) under *N* = 70. The scratch model exhibits a mode‐collapse pattern, with predictions concentrated on a single class and a weak diagonal structure, confirming convergence to a trivial solution. In contrast, the SSL‐initialized model maintains a distinct diagonal and distributed predictions, reflecting preserved discriminative capability and effective suppression of collapse symptoms.

Confusion matrices further reveal the characteristic manifestation of optimization collapse under extreme few‐shot supervision (Figure 7). In the worst‐performing scratch‐trained run, the model converges to a trivial solution, with predictions overwhelmingly concentrated in a single class (typically SR). This results in a pronounced vertical pattern in the confusion matrix and a severely weakened diagonal structure, consistent with the early training stagnation observed in Section 3.2.

In contrast, the best‐performing SSL‐NoAug run exhibits a clear diagonal structure and a more balanced prediction distribution across classes. This alignment between process‐level evidence (delayed best validation epochs) and outcome‐level evidence (confusion matrix structure) indicates that SSL initialization suppresses the single‐class prediction bias induced by data scarcity. Such behavior is in line with prior analyses showing that improved representation quality mitigates mode collapse and degenerate solutions in low‐data regimes.

### 3.6 Model Interpretability: Overcoming Shortcut Learning to Restore Clinical Logic

**FIGURE 8:**
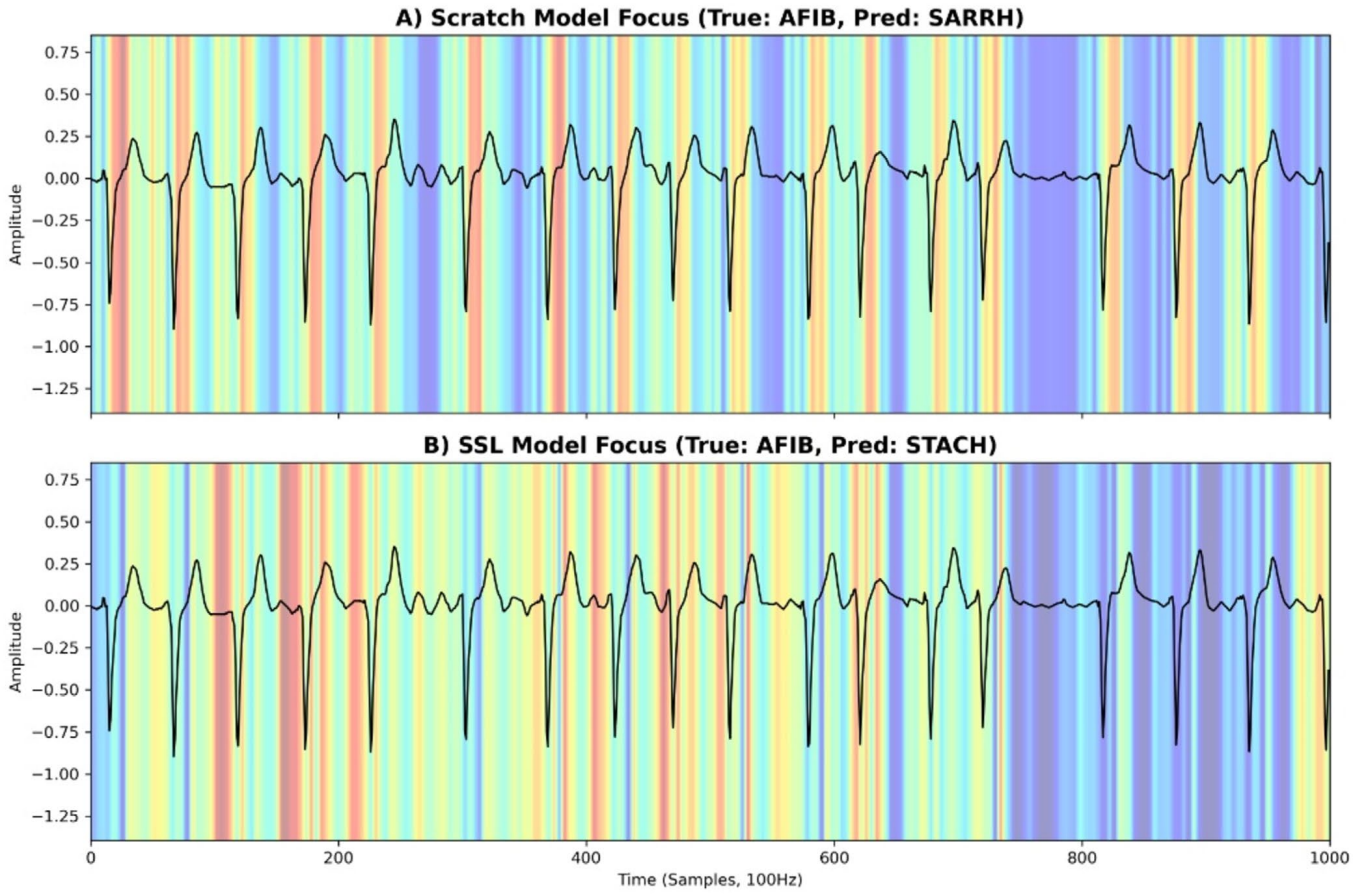
1D Grad‐CAM visualization of model attention on a representative Atrial Fibrillation (AFIB) case. (A) Scratch Model: Under extreme few‐shot conditions (*N* = 70), the supervised model suffers from “shortcut learning” and collapses into a naive peak‐detector. It allocates disproportionately high attention (weight = 0.64) to the non‐diagnostic QRS peak, failing to capture the true pathological hallmarks of AFIB. (B) SSL Model (DNoAug): The SSL‐initialized model successfully recovers clinical diagnostic logic. It intelligently attenuates its focus on the QRS peak (weight = 0.40) and distributes its attention across the inter‐beat baseline intervals to search for fibrillatory f‐waves and evaluate the absence of P‐waves. This quantitative shift demonstrates that self‐supervised pre‐training guides the model to learn genuine cardiac electrophysiology rather than overfitting to high‐amplitude artifacts.

To verify whether the performance gain of SSL translates to clinically trustworthy decision‐making, we applied 1D Grad‐CAM to visualize and quantify the spatial focus of the models. Figure 8 illustrates a representative case of Atrial Fibrillation (AFIB). Clinically, AFIB is diagnosed by the absence of P‐waves and the presence of fibrillatory waves on the baseline, rather than the morphology of the QRS complex. Our quantitative analysis reveals that the collapsed scratch‐trained model acts as a naive peak detector, inappropriately allocating high attention weight (0.64) to the non‐diagnostic QRS peak. In contrast, the SSL‐initialized model (D_NoAug_) intelligently attenuates its focus on the QRS peak (0.40) and distributes its attention across the inter‐beat intervals to evaluate fibrillatory activity. This visualization confirms that self‐supervised pre‐training not only stabilizes the optimization trajectory but also restores clinical interpretability, ensuring that the model learns genuine cardiac electrophysiology rather than overfitting to high‐amplitude noise under extreme data scarcity.

## 4 Discussion

### 4.1 The Core Challenge: Early Collapse, Not Low Accuracy

This study deliberately focuses on an extreme few‐shot setting (*N* = 70, 14 samples per class) that reflects realistic clinical constraints. Under this regime, the dominant limitation is not the conventional notion of reduced generalization due to data scarcity, but a more fundamental issue of trainability. Scratch‐trained supervised models frequently collapse at the very early stages of training, characterized by best validation epochs occurring at or near the first epoch, highly biased prediction distributions, and the inability to make further progress thereafter. Unlike gradual overfitting, we observe a distinct failure mode characterized by stereotyped early‐training dynamics and consistent error behavior across seeds; we therefore treat it as a systematic optimization/training failure rather than incidental low performance. This framing aligns with prior theory emphasizing that modern deep learning behavior cannot always be reduced to a simple overfitting narrative, and that nonconvex optimization and initialization sensitivity can induce qualitatively different regimes of training and generalization behavior (Belkin et al. 2019).

This distinction is critical from a methodological perspective, as it reframes the problem from “how much performance is lost with fewer labels” to “whether learning reliably occurs at all.” The training‐dynamics evidence in Figures 3, 4 demonstrates that the high collapse rates observed for scratch training and augmentation‐only baselines indicate that models often fail to enter an effective learning phase under extreme supervision. Similar instability and early convergence to degenerate solutions have been reported in low‐data deep learning settings, where optimization behavior rather than model capacity becomes the primary bottleneck.

### 4.2 SSL Improves Trainability by Reshaping Optimization Paths

The most important contribution of SSL in this work is not a marginal increase in mean performance, but a qualitative change in optimization behavior. Pretraining on large collections of unlabeled ECG signals yields smoother and more transferable representation manifolds, shifting the downstream task from constructing features from sparse supervision to aligning decision boundaries within an already informative embedding space.

This mechanism explains two key observations. First, SSL initialization dramatically suppresses early collapse: in the D (SSL, NoAug) configuration, the collapse rate decreases from 66.7% to 0%, and the best validation epoch shifts substantially later (Figures 2 and 3), indicating sustained learning rather than premature stagnation. Second, inter‐seed variance contracts sharply under SSL initialization, suggesting that the training process avoids regions of the loss landscape that are highly sensitive to initialization and stochastic perturbations. From an optimization standpoint, this suggests that SSL provides a more favorable initialization that improves trainability and stabilizes the early (transient) phase of learning, consistent with prior analyses showing that well‐designed initialization can be crucial for successfully training deep networks, whereas poor initialization can lead to training failure (Sutskever et al. 2013).

Behavioral evidence from confusion matrices (Figure 6) further supports this interpretation. In collapse cases, the model converges to a near‐constant predictor dominated by a single class, whereas SSL‐initialized models recover a clear diagonal confusion structure and more distributed predictions. This supports our claim that SSL mitigates class‐wise degeneration rather than merely inflating performance through a dominant category. More broadly, this emphasis on diagnosing failure modes is aligned with prior evidence that standard ERM training can exhibit undesirable behaviors (e.g., memorization and instability), and that principled regularization/augmentation schemes can alleviate such issues (Zhang et al. 2017). Similar links between representation quality and avoidance of degenerate solutions have been noted in prior studies on mode collapse and biased predictions under limited supervision.

### 4.3 When It Fails: Why Strong Augmentation Destabilizes Extreme Few‐Shot Learning

The ablation results provide a clear answer to a natural reviewer question: can stronger regularization or augmentation alone solve the problem? Under *N* = 70, the answer is no. Augmentation‐only training exhibits collapse behavior comparable to scratch training, and even after SSL initialization, enabling strong downstream augmentation reintroduces instability.

A plausible explanation is that, when supervision is extremely limited, gradient updates rely on very weak signals from each mini‐batch. Strong augmentations increase input variance and effectively dilute the already scarce supervisory information, pushing optimization toward degenerate or oscillatory solutions. Data augmentation is widely used as a powerful way to improve generalization and mitigate overfitting, but its benefits are not guaranteed in very limited‐data regimes and may even exacerbate overfitting or fail to address biases inherent to small datasets (Shorten and Khoshgoftaar 2019).

Class‐wise analysis further clarifies the boundary conditions. Certain rhythms (e.g., STACH) are intrinsically difficult to separate using short 10‐s segments with limited samples, leading to near‐zero F1 under scratch training. While SSL improves separability across multiple classes, the gains remain bounded, highlighting constraints imposed by intra‐class variability, temporal window length, and sample coverage. Explicitly acknowledging these limits strengthens the credibility of the conclusions.

### 4.4 Clinical Implications: Reproducibility Over Peak Scores

In real‐world clinical workflows, the bottleneck for ECG AI deployment is rarely model architecture, but rather the availability of high‐quality, scalable annotations. A common starting point for new hospitals, devices, or clinical settings is a dataset with only dozens of reliable labels. In such contexts, the most critical failure is not a modest drop in accuracy, but non‐reproducible training behavior, where identical data and configurations yield radically different outcomes depending on random initialization.

From this perspective, the primary value of SSL in extreme few‐shot ECG classification lies in transforming training from a high‐risk, unpredictable process into a reproducible and controllable pipeline. By substantially reducing collapse rates and inter‐run variability, SSL initialization improves not only performance but also deployability and trustworthiness, which are essential for regulatory approval and ongoing quality management in clinical AI systems. Similar arguments emphasizing robustness and reproducibility as prerequisites for clinical impact have been articulated in high‐impact medical AI literature.

### 4.5 Limitations and Clinical Perspectives

Several limitations should be acknowledged and interpreted as boundary conditions rather than shortcomings. Crucially, a notable limitation of this study is the artificial construction of the “extreme few‐shot” scenario using clinically common arrhythmias (e.g., AFIB, SR), which are typically abundant in real‐world databases. This design was methodologically necessary to establish a controlled “proof‐of‐concept” with a measurable upper‐bound baseline (*N* = 300). Without this controlled setting, it would be impossible to causally isolate the mechanism of optimization collapse. However, the true clinical value of our proposed SSL stabilization framework lies in its application to genuinely rare cardiovascular conditions, such as cardiac amyloidosis or inherited channelopathies, where obtaining large annotated cohorts is practically impossible. By validating the anti‐collapse mechanism and clinical interpretability in this controlled simulation, our study lays the methodological foundation for deploying SSL to tackle real‐world diagnostic challenges of rare ECG manifestations.

Furthermore, on a technical level, the findings are derived from a single public dataset (PTB‐XL), a single task formulation (five single‐label rhythms, 10 s segments at 100 Hz), and a single model family (lightweight CNN–Transformer). The few‐shot splits were designed to isolate training stability mechanisms and do not yet address patient‐wise separation, cross‐device or cross‐center domain shifts, or more realistic multi‐label rhythm scenarios. Finally, external validation on independent datasets was not performed. Accordingly, the current conclusions should be interpreted as evidence that SSL markedly improves trainability and reproducibility within the extreme few‐shot regime of PTB‐XL, while broader generalization remains to be evaluated.

### 4.6 Future Directions

Future work can extend this study with minimal but high‐impact additions. First, external validation or transfer experiments on another public ECG dataset would test whether the stability advantages of the SSL‐NoAug configuration generalize beyond PTB‐XL. Second, enforcing strict patient‐wise splits would further reduce leakage and better reflect deployment conditions. Third, extending the task to multi‐label rhythm classification or a larger set of rhythm categories would allow evaluation of SSL under more realistic long‐tailed clinical distributions, while reusing the same stability metrics (Macro F1, collapse rate, best epoch) for consistency.

## 5 Conclusion

This study presents and validates a stability‐centered methodology for extreme few‐shot ECG rhythm classification. Using the PTB‐XL dataset, we formulated a five‐class rhythm task and deliberately constrained supervised training to an extreme few‐shot regime (*N* = 70). Our results demonstrate that, under this setting, scratch‐based supervised learning does not merely exhibit reduced performance but instead suffers from systematic training failure, characterized by early collapse to degenerate solutions, best validation epochs occurring at the first iteration, highly biased predictions, and amplified inter‐seed variability.

In contrast, self‐supervised contrastive pretraining provides a substantially healthier initialization. When combined with conservative fine‐tuning without downstream augmentation, SSL reduces the collapse rate from 66.7% to 0%, markedly improves Macro F1, and nearly eliminates run‐to‐run variance. Additional ablation analyses show that strong downstream augmentation cannot substitute for representation pretraining and may even introduce further instability under extreme label scarcity. Class‐wise and confusion‐matrix analyses further support the proposed mechanism: the benefits of SSL arise from restoring multi‐class separability and suppressing single‐class prediction bias, rather than inflating scores for a dominant class.

Taken together, this work provides a reproducible training strategy for ECG classification under severe label scarcity and introduces a reusable framework for diagnosing training stability using process‐level metrics. By prioritizing trainability and run‐to‐run reproducibility over single‐run peak scores, our findings align with broader concerns that medical AI has been largely enabled by labeled big data yet remains constrained by bias, privacy/security, and transparency limitations, which directly affect how such systems can be trusted and operationalized in practice (Topol 2019). More generally, our emphasis on stability diagnostics and robust reporting is consistent with evidence from benchmark research showing that over‐reliance on repeatedly used test sets can mask true generalization, as models exhibit non‐trivial accuracy drops on carefully reconstructed new test sets. Together, these considerations motivate evaluation protocols that treat reproducibility and failure‐mode characterization as first‐class objectives when translating ECG models into low‐resource clinical workflows (Recht et al. 2019).

## Author Contributions

**LiuPing Zeng:** conceptualization, methodology, formal analysis, visualization, and writing – original draft. **JingMei Pan:** conceptualization, methodology, supervision, and writing – review and editing. **YangJie Lu:** data curation, investigation, formal analysis, and validation. **Xian Pan:** investigation, validation, and writing – review and editing. All authors read and approved the final manuscript.

## Conflicts of Interest

The authors declare no conflicts of interest.

## Data Availability

All data analyzed in this study were obtained from the publicly available PTB‐XL dataset (version 1.0.3) hosted on PhysioNet. No new human participant data were collected for this study. The PTB‐XL dataset record and its associated documentation are publicly accessible via PhysioNet and are cited in the References section.
